# Long‐term renal follow up of preterm neonates born before 35 weeks of gestation

**DOI:** 10.1111/ped.14004

**Published:** 2019-12-22

**Authors:** Akiyoshi Horie, Yasuhiro Abe, Daisuke Koike, Tomohiro Hirade, Akiyoshi Nariai, Tomoko Ito, Fumihide Katou

**Affiliations:** ^1^ Division of Pediatrics Shimane Prefectural Central Hospital Himebara, Izumo, Shimane Japan; ^2^ Division of Neonatology Shimane Prefectural Central Hospital Himebara, Izumo, Shimane Japan

**Keywords:** chronic kidney disease, estimated glomerular filtration rate, low‐birthweight, premature birth, small for gestational age

## Abstract

**Background:**

The hypothesis of the Developmental Origins of Health and Disease states that environmental factors during fetal and infantile life are risk factors for some chronic diseases in adulthood. Few studies, however, have confirmed this hypothesis early in childhood. Therefore, we assessed how premature birth and low‐birthweight (LBW) affect the renal function of Japanese children.

**Methods:**

This retrospective study surveyed 168 patients who were born before 35 weeks of gestation and were cared for at the present neonatal intensive care unit. Follow‐up duration was >2 years. Serum creatinine (sCr) and estimated glomerular filtration rate (eGFR) recorded in medical records were reviewed.

**Results:**

The eGFR at 2 years of age was significantly correlated with birthweight and gestational age (*P *<* *0.01). Approximately 10.7% of the children had low eGFR (<90 mL/min/1.73 m^2^) without clinical symptoms or abnormal urine examination. These children had high sCr on day 7 after birth (*P *<* *0.01) and delayed recovery of these levels during the first month after birth.

**Conclusion:**

Premature gestational age and LBW directly affect renal function in young children. High sCr on day 7 after birth is a risk factor for chronic kidney disease in children. Careful follow up of renal function is therefore required for premature infants and infants with LBW beginning in early childhood to prevent renal dysfunction.

The prognosis of preterm infants has improved with developments in neonatal medicine. According to the hypothesis of the Developmental Origins of Health and Disease (DOHaD),^1^ environmental factors during fetal and infant life are risk factors for some chronic diseases, and the prevalence of these chronic diseases has been increasing.^2^, ^3^

Glomeruli are fewer in number and less mature in preterm infants than in full‐term infants,^4^, ^5^ and this difference could be a risk factor for hypertension and renal dysfunction later in adulthood.^2^, ^3^, ^6^ There is little information, however, on the renal function of preterm infants during childhood. Renal inulin clearance is an accurate method of measuring glomerular filtration rate (GFR), but collection of urine samples from this patient population is difficult. Therefore, it is difficult to evaluate the renal function of these children. In 2011, Uemura *et al*. estimated the GFR of Japanese children using serum creatinine (sCr) on enzymatic methods and age, body length, and sex based on the Schwartz formula.^7^ Their protocol established a very useful and simple method of evaluating estimated GFR (eGFR) during childhood in Japan.

Glomerulogenesis begins during the fifth week of gestation in a developing fetus and is completed at approximately 36 weeks of gestation. The exact number of nephrons that develop during glomerulogenesis differs depending on race and sex. In Japanese fetuses, glomerulogenesis is completed at approximately 35 or 36 weeks of gestation.^5^ Generally, GFR reaches an adult level by 2 years of age.

Preterm birth and low‐birthweight (LBW) have been shown to affect sCr and renal function during the neonatal period,^8^ but little is known about the renal function of preterm infants during early childhood. The aims of this study were therefore to understand the renal function of preterm neonates born before 35 weeks of gestation and to identify potential risk factors for renal dysfunction in Japanese children older than 2 years. This article is a follow‐up report based on a study first reported in *Japanese Journal of Pediatric Nephrology* in Japanese.^9^ The authors have received approval for publication from the editor of the primary publication journal (*JJPN*).

## Methods

### Patients

Data used for this study were retrospectively collected via medical records from the electronic database program at Shimane Prefectural Central Hospital. We collected medical records of infants born before 35 weeks of gestation who were admitted to the neonatal intensive care unit (NICU) between 1 April 1999 and 31 March 2016.

Medical records were reviewed for gestational age, bodyweight and length at birth, sex, Apgar score at 1 and 5 min of age, and use of antimicrobial agents, steroid, or indomethacin. sCr data available between days 1 and 7 and those on days 14, 21, and 30 after birth were collected retrospectively for each patient. Creatinine was measured using an enzymatic assay. When the infant was older than 2 years, bodyweight, body length, and renal function, which were indicated by the eGFR, were reviewed.

Exclusion criteria were the following conditions that developed while the patient was in the NICU: congenital urinary tract malformation on ultrasonography, major congenital anomalies, congenital heart disease, or acute kidney dysfunction. We excluded patients older than 2 years with abnormal urinary qualitative test and those treated with angiotensin‐converting enzyme inhibitors or angiotensin receptor inhibitors. Additionally, we excluded sCr data associated with urinary tract infections and dehydration collected in the emergency department.

All patients included in this study were born before 35 weeks of gestation and followed up until they were aged >2 years. These patients were divided into two groups based on eGFR: a low eGFR group (eGFR < 90 mL/min/1.73 m^2^ that persisted without clinical symptoms or abnormal urine examination) and a control group (eGFR > 90 mL/min/1.73 m^2^).

### Statistical analysis

Mann–Whitney *U*‐test was used to compare bodyweight, body length, and sCr on different days. Spearman rank‐order test was used to determine the correlations between eGFR, gestational weeks, and birthweight. Data are expressed as mean ± SD. The chi‐squared test was used to determine the correlation between the use of indomethacin and aminoglycoside agents and the number of infants who were small for gestational age (SGA). All analyses were conducted using Microsoft Excel (Microsoft Corporation, WA, USA) and Ystat 2000 (Igakutosho‐shuppan, Saitama, Japan). In all analyses, *P *<* *0.05 was considered statistically significant.

### Ethics

All procedures performed in studies involving human participants were in accordance with the ethics standards of the Ethical Review Board at Shimane Prefectural Central Hospital, at which the studies were conducted (approval number R16‐052) and with the 1964 Helsinki declaration and its later amendments or comparable ethics standards.

## Results

**Figure 1:**
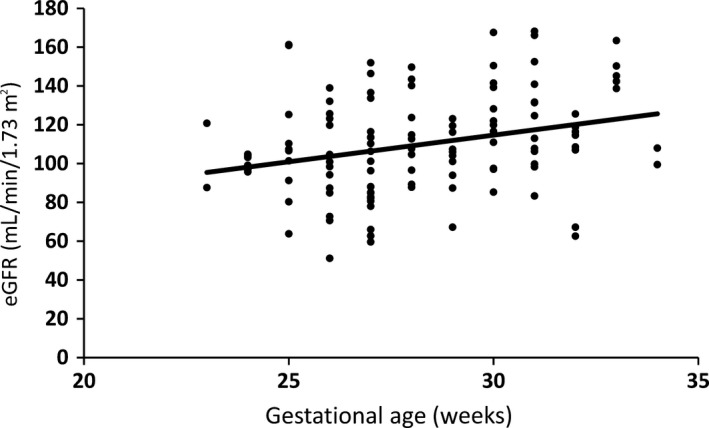
Relationship between eGFR at age 2 years and gestational weeks for infants born before 35 weeks of gestation. (*n* = 119)

**Figure 2:**
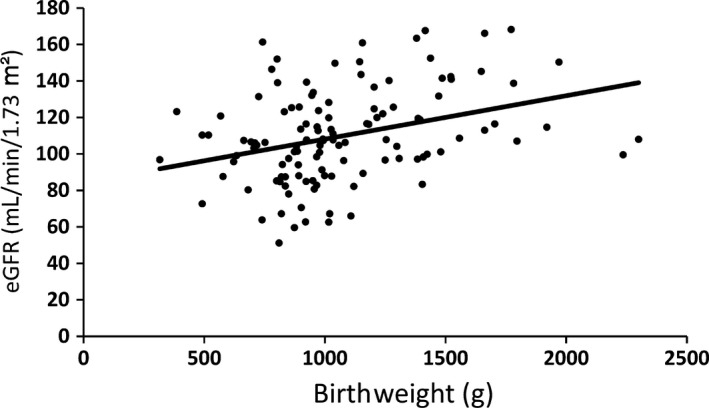
Relationship between eGFR at age 2 years and birth weight of infants born before 35 weeks of gestation. (*n* = 119)

During the study period, 6,977 neonates were hospitalized in the neonatal department and 499 preterm neonates born before 35 weeks of gestation were admitted to the NICU. Of these 499 neonates, 168 met the inclusion criteria and were followed up after 2 years of age. Demographics of the infants are summarized in Table 1. Bodyweight, height, sCr, and eGFR were obtained. At 2 years of age, bodyweight, height, and sCr were determined for 119 of the 168 neonates (68 boys, 51 girls). Mean weight was 10.1 ± 1.6 kg, mean height was 80.7 ± 4.7 cm, mean sCr was 0.25 ± 0.06 mg/dL, and mean eGFR was 109.8 ± 25.7 mL/min/1.73 m^2^. There were no significant differences in sex, SGA, or use of indomethacin and aminoglycoside agents (Table 2), but eGFR at 2 years of age was significantly and positively correlated with birthweight (*P *<* *0.01; *n *=* *119; rs = 0.30803; *y* = 2.75*x*+32.25; Fig. 1) and gestational age (*P *<* *0.01; *n *=* *119; rs = 0.34666; *y* = 0.02*x*+84.34; Fig. 2). Although not significant, eGFR at 3 and 4 years of age also had positive correlations with birthweight and gestational age (data not shown). Mean eGFR at 3 years of age was 115.1 ± 22.5 mL/min/1.73 m^2^ (*n *=* *31), and at 4 years of age it was 108.0 ± 24.8 mL/min/1.73 m^2^ (*n *=* *23).

**Table 1:** Demographics of born before 35 weeks of gestation (*n* = 168)

	Range (median) or n (%)
Age (years)	2–15 (5)
M:F	82:86
Birthweight (g)	
<1,000	73 (43.5)
1,000–1,500	76 (45.2)
>1,500	19 (11.3)
Gestational age (weeks + days)	
<28 + 0	63 (37.5)
≥28 + 0	105 (62.5)
SGA	57 (33.9)
Indomethacin	44 (26.2)
Antimicrobial agent	151 (89.9)
Aminoglycoside	109 (64.9)

SGA, small for gestational age.

**Table 2:** eGFR at age 2 years in subjects born before 35 weeks of gestation (n = 119)

	n or mean ± SD		*P*‐value
M/F	68/51		
SGA at birth	+	−	
*n*	33	86	
eGFR (mL/min/1.73 m^2^)	114.9 ± 22.9	109.9 ± 25.9	>0.05
Indomethacin use in NICU	+	−	
*n*	30	89	
eGFR (mL/min/1.73 m^2^)	107.7 ± 19.9	112.8 ± 26.9	>0.05
Aminoglycoside agent use in NICU	+	−	
*n*	76	43	
eGFR (mL/min/1.73 m^2^)	113.4 ± 24.2	104.9 ± 27.6	>0.05

eGFR, estimated glomerular filtration rate; NICU, neonatal intensive care unit; SGA, small for gestational age.

For the 168 patients who were followed up after 2 years of age, 18 (10.7%) had persistently low eGFR (<90 mL/min/1.73 m^2^; low eGFR group). Low eGFR, however, was not necessarily associated with clinical symptoms such as malaise or anemia described in the medical records of these patients. The demographics of these patients are summarized in Tables 3,4. There were no significant differences in sex, history of patent ductus arteriosus (PDA) ligation, or use of indomethacin, steroid in the postnatal period, or aminoglycoside agents. Interestingly, in the low eGFR group, all infants born after 30 weeks of gestation were SGA. For 119 preterm neonates born after 30 weeks of gestation in this study, 29 were SGA, and, of these, four had low eGFR; 90 infants were not SGA, and of these one infant had low eGFR (*P *<* *0.05).

**Table 3:** Demographics of the low eGFR group (<90 mL/min/1.73 m^2^) at >2 years

	*n *= 18 (M:F, 9:9)Range (median), mean ± SD or n (%)
Age (years)	4–16 (6)
eGFR (mL/min/1.73 m^2^)	79.2 ± 9.2
Gestational age (weeks + days)	
<28 + 0	6/70 (8.6)
≥28 + 0	12/102 (11.8)
Birthweight (g)	
<1,000	11/74 (14.9)
1,000–1,500	7/77 (9.1)
>1,500	0/21
Indomethacin	9/18 (50.0)
PDA ligation	3/18 (16.7)
Aminoglycoside	11/18 (61.1)
Steroid	9/18 (50.0)

eGFR, estimated glomerular filtration rate; PDA, patent ductus arteriosus.

**Table 4:** Demographics of the low eGFR group (<90 mL/min/1.73 m^2^) during infancy

Patient ID no.	Gestation (weeks + days)	Birthweight (g)	SGA	Sex	Apgar score (1/5 min)	Aminoglycoside	Steroid	Indomethacin	PDA ligation
1	23 + 5	578		M	1/3	○	○	○	○
2	25 + 5	682	○	M	5/7	○	○	○	
3	26 + 0	492	○	F	1/6	○			
4	26 + 1	845		F	4/5	○			
5	27 + 1	874		M	6/7	○	○	○	
6	27 + 1	920		F	6/9	○			
7	27 + 6	836	○	F	5/6		○	○	
8	28 + 1	934	○	M	5/8		○	○	○
9	28 + 1	1058		M	3/‐	○		○	
10	28 + 3	1158		F	9/9	○			
11	28 + 4	1100		F	6/9	○		○	
12	29 + 3	836	○	M	2/−	○	○	○	
13	29 + 5	1480		M	3/−		○		
14	30 + 0	1390		F	5/7		○	○	○
15	30 + 3	996	○	F	1/8		○		
16	30 + 6	686	○	F	6/9	○		○	
17	31 + 5	1166	○	M	7/9				
18	32 + 3	1016	○	M	8/9				

eGFR, estimated glomerular filtration rate; PDA, patent ductus arteriosus; SGA, small for gestational age.

In the low eGFR group, sCr, body height, and eGFR were obtained for 17 of the 18 patients at 2 years of age. There were significant differences (*P *<* *0.05) in bodyweight, height, and eGFR between the low eGFR and control groups (weight, 9.0 ± 1.4 kg; height, 76.7 ± 3.9 cm; eGFR, 79.2 ± 9.2 mL/min/1.73 m^2^, respectively), but there was no significant difference in sCr (0.33 ± 0.06 mg/dL).

**Figure 3:**
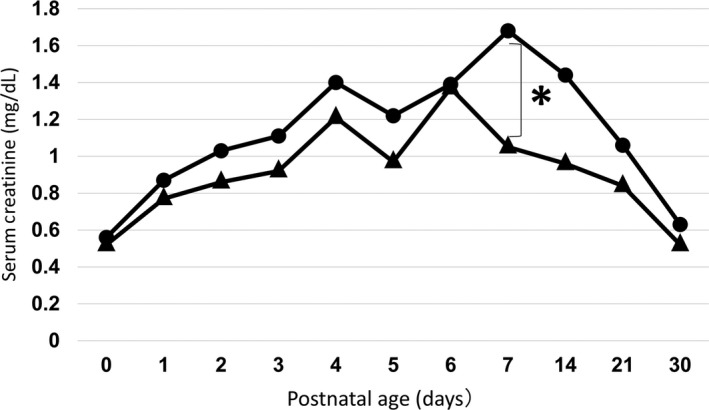
Serum creatinine during the first days of life in infants born before 35 weeks of gestation in the (●) low estimated glomerular filtration rate group and the (▲) control group. *P < 0.01 at day 7 of life.

sCr level during infancy is shown in Figure 3. The control group had increased sCr on day 4 of life. These levels reached a plateau between days 4 and 6 of life, and then decreased thereafter. In the low eGFR group, however, sCr persistently increased until day 7 of life. Furthermore, there was a significant difference between the low eGFR and control groups on day 7 of life (1.68 ± 0.71 mg/dL vs 1.05 ± 0.55 mg/dL, respectively; *P *<* *0.01; effect size, 1.11; power [1‐β], 0.93). On days 14, 21, and 30 of life, the recovery of sCr was delayed. In addition, there were significant differences on Mann–Whitney *U*‐test (*P *<* *0.05), but the effect size and power (1‐β) were small.

## Discussion

Based on the DOHaD hypothesis, few premature glomeruli form in preterm infants and those with LBW through glomerular hyperfiltration. Glomerular hyperfiltration can be a predictor of glomerular hypertension, which leads to adverse cardiovascular events, secondary glomerulosclerosis, and chronic kidney disease later in adulthood.^2^, ^6^, ^10^

Low‐birthweight increases the risk of low GFR and renal dysfunction in school‐age children,^11^, ^12^ but little is understood regarding renal function during early childhood, or regarding the relationship between gestational weeks at birth and birthweight for children in Japan and other countries.

It is uncertain when eGFR reaches an adult level in preterm and LBW infants. There were significant positive correlations, however, between eGFR, birthweight, and gestational age at 2 years of age. Moreover, eGFR at 3 and 4 years of age also showed a similar tendency to each other in this study. This suggests that preterm birth and LBW affect renal function during early childhood.

Eighteen of the 168 patients (10.7%) who were followed up until >2 years of age had persistently low eGFR (<90 mL/min/1.73 m^2^). It was also observed, however, that the patients had no clinical symptoms and did not have any major health problems. Medical examinations yielded no abnormalities in urinary qualitative test results for any of the 18 patients. Additionally, sCr was abnormal in six of the 18 patients at all ages. At 60 months of age, children with very LBW have been shown to have lower eGFR than those with normal birthweight, but there is no significant difference in sCr.^11^, ^13^ Therefore, it can be concluded that eGFR is needed to evaluate renal function in infants with normal sCr and short stature. Moreover, the Low Birth Weight and Nephron Number Working Group has recommended that follow up of LBW and preterm infants include annual blood pressure, renal ultrasound, and urinalysis from early childhood.^3^

Furthermore, in the present study, patients with persistently low eGFR at >2 years of age had significantly high sCr on day 7 of life and delayed recovery of sCr level. Some parameters have been reported to affect renal function during the neonatal period, such as fractional excretion of sodium, gestational weeks, birthweight, mechanical ventilation, drugs, and maternal morbidity.^3^, ^14^, ^15^, ^16^ Surveys, such as those performed by Auron and Mhanna, noted elevated sCr in very LBW infants on day 2 of life.^8^ This high level decreased postnatally and reached a plateau between days 4 and 6 of life. They also found that sCr does not decrease significantly during the first 6 days of life in infants born before 29 weeks of gestation and with birthweight <1,000 g.^8^ This further suggests that the postnatal development of renal function is related to gestational age and is slower in premature infants.

In the low eGFR group, all infants born after 30 weeks of gestation were SGA at birth. SGA could result from intrauterine growth restriction caused by factors such as placental insufficiency, undernutrition, or malnutrition. Therefore, infants fail to reach their genetic growth potential. SGA status of preterm infants has also been shown to affect the increase of apoptotic cells in the fetal kidney and to decrease renin and angiotensinogen.^17^ Furthermore, the number and formation of nephrons are compromised, leading to lower fractional sodium excretion and GFR.^2^, ^3^, ^12^, ^18^ Specifically, Drougia *et al*. found that SGA infants born before 34 weeks of gestation had significantly smaller kidneys at 24 months of age.^19^ Therefore, it can be hypothesized that SGA status is another factor that contributes to renal dysfunction.

### Limitations

There were many limitations in this study. We conducted a retrospective review of patient medical records; therefore, we did not have prenatal medical records available for review. Any prenatal exposure to nephrotoxic medications could have altered the present results. Precise data regarding dosage and time of exposure to drugs, such as indomethacin, steroid, or aminoglycoside agents, were not accessible for review, and the potential effects of these agents on renal function could not be assessed. Furthermore, we did not exclude patients using conventional mechanical ventilation. Any form of mechanical ventilation could have resulted in increased intrathoracic pressure, which may impair venous return and renal function. sCr data of newborns were collected retrospectively; therefore, we could not measure creatinine clearance, cystatin C, or urinary β‐2 microglobulin. Additionally, we could not measure other biological parameters of renal function that could be used to correlate with gestational age. The lack of data regarding fluid intake, water balance, and hypernatremic dehydration during the first week of life also limited the interpretation of sCr data.

Clinical data obtained during the follow‐up period after discharge from the NICU, such as medication prescribed by physicians at other hospitals and environmental factors, were not assessed, but they could have influenced changes in sCr level in young children. Therefore, further prospective studies are necessary to explore the development of renal function in preterm infants and to determine whether multifactorial events occurring early during postnatal life could have long‐term consequences on renal outcomes later in life.

In conclusion, in the present patients at 2 years of age, there were significant positive correlations between eGFR, gestational age, and birthweight for preterm infants born before 35 weeks of gestation. This means that preterm birth and LBW affect renal function not only during adulthood but also during early childhood.

More than 10% of the present patients had persistently low renal function without any symptoms. These patients often had short stature compared with children born at full term. Therefore, even if sCr was not increased, data regarding sCr were not sufficient to evaluate renal function; these data, however, in addition to data on height, are necessary to estimate GFR.

During the neonatal period, high sCr at day 7 after birth and delayed recovery of sCr level were risk factors for renal dysfunction. In addition, for fetuses at 30 weeks of gestation, SGA was a risk factor for renal dysfunction. Therefore, more careful follow‐up is needed to prevent renal dysfunction in these patients.

## Disclosure

The authors declare no conflict of interest.

## Author contributions

A.H. and F.K. designed the study; A.H., Y.A., D.K., T.H., A.N., T.I., and F.K. collected and analyzed the data; A.H. performed the statistical analysis and drafted the manuscript; A.N. and F.K. critically reviewed the manuscript and supervised the entire study process. All authors read and approved the final manuscript.
